# Supplementary material for: Validation of Inflammatory Prognostic Biomarkers in Pleural Mesothelioma
Source: Cancers (Basel). 2023 Dec 24;16(1):93. doi: 10.3390/cancers16010093 (PMC10778470; doi:10.3390/cancers16010093)
Supplement: Supplementary file 1 [file cancers-16-00093-s001.zip › cancers-2767388-supplementary.pdf]

Supplementary Table S1. Partaking Departments.

Department	Number of patients	Ethics committee
Gazi University, School of Medicine, Department of Thoracic Surgery, Ankara, Turkey	41	Gazi University Clinical Research Ethics Committee 19/11/2022
Karl Landsteiner University of Health Sciences, Department of General and Thoracic Surgery, University Hospital Krems, Austria	38	Commission for Scientific Integrity and Ethics of the Karl Landsteiner Private University 1047/2021
IRCCS Azienda Ospedaliero Universitaria di Bologna, Division of Thoracic Surgery, Italy	12	Comitato Etico Area Vasta Emilia Centro CE AVEC 340/2022/Oss/AOUBo
Barts Thorax Centre, St Bartholomew’s Hospital, Barts Health NHS Trust, London, UK	7	Clinical Ethics Committee, The Royal Hospital NHS Trust, no. 2520
